# Supplementary material for: MetaRibo-Seq measures translation in microbiomes
Source: Nat Commun. 2020 Jun 29;11:3268. doi: 10.1038/s41467-020-17081-z (PMC7324362; doi:10.1038/s41467-020-17081-z)
Supplement: Supplementary file 10 — Supplementary Data 7 [file 41467_2020_17081_MOESM10_ESM.zip › File2/Confidence_High_Taxonomy/181175_out.krona.html]

Javascript must be enabled to view this page.

members
magnitude
magnitudeUnassigned
count
unassigned
taxon
rank

181175\_out

11

2
superkingdom
11

11
1239
phylum

3
186801
class

3
186802
order

31979
family
3

3
genus
1485

59620

SRS097958\_contig\_number\_25286SRS104485\_contig\_number\_3362SRS142503\_contig\_number\_38962
species
3

species

SRS014948\_contig\_number\_16720SRS015578\_contig\_number\_18300SRS019068\_contig\_number\_contig-100\_2290.414492SRS052697\_contig\_number\_39086SRS063985\_contig\_number\_16493SRS077231\_contig\_number\_contig-100\_30363.30364SRS144506\_contig\_number\_25100SRS148159\_contig\_number\_contig-100\_876.321984
2292898
8
